# Feature tracking measurement of dyssynchrony from cardiovascular magnetic resonance cine acquisitions: comparison with echocardiographic speckle tracking

**DOI:** 10.1186/1532-429X-15-95

**Published:** 2013-10-17

**Authors:** Toshinari Onishi, Samir K Saha, Daniel R Ludwig, Tetsuari Onishi, Josef J Marek, João L Cavalcante, Erik B Schelbert, David Schwartzman, John Gorcsan

**Affiliations:** 1 The University of Pittsburgh, 200 Lothrop Street, Pittsburgh, PA, USA; 2 Sundsvall Hospital, Sundsvall, Sweden

**Keywords:** Strain, Dyssynchrony, Echocardiography, Cardiovascular magnetic resonance

## Abstract

**Background:**

Analysis of left ventricular (LV) mechanical dyssynchrony may provide incremental prognostic information regarding cardiac resynchronization therapy (CRT) response in addition to QRS width alone. Our objective was to quantify LV dyssynchrony using feature tracking post processing of routine cardiovascular magnetic resonance (CMR) cine acquisitions (FT-CMR) in comparison to speckle tracking echocardiography.

**Methods:**

We studied 72 consecutive patients who had both steady-state free precession CMR and echocardiography. Mid-LV short axis CMR cines were analyzed using FT-CMR software and compared with echocardiographic speckle tracking radial dyssynchrony (time difference between the anteroseptal and posterior wall peak strain).

**Results:**

Radial dyssynchrony analysis was possible by FT-CMR in all patients, and in 67 (93%) by echocardiography. Dyssynchrony by FT-CMR and speckle tracking showed limits of agreement of strain delays of ± 84 ms. These were large (up to 100% or more) relative to the small mean delays measured in more synchronous patients, but acceptable (mainly <25%) in those with mean delays of >200 ms. Radial dyssynchrony was significantly greater in wide QRS patients than narrow QRS patients by both FT-CMR (radial strain delay 230 ± 94 vs. 77 ± 92* ms) and speckle tracking (radial strain delay 242 ± 101 vs. 75 ± 88* ms, all *p < 0.001).

**Conclusions:**

FT-CMR delivered measurements of radial dyssynchrony from CMR cine acquisitions which, at least for the patients with more marked dyssynchrony, showed reasonable agreement with those from speckle tracking echocardiography. The clinical usefulness of the method, for example in predicting prognosis in CRT patients, remains to be investigated.

## Background

Cardiac resynchronization therapy (CRT) has had a major impact on many heart failure patients with depressed ejection fraction conferring symptomatic relief and survival benefit [1-3]. Although QRS width and morphology are used as the primary selection criteria for CRT, QRS the finding of baseline mechanical dyssynchrony has important prognostic utility [4-7] CRT response remains variable with approximately one-third of patients not responding [1-3]. Accordingly, there has been a building level of interest to quantify myocardial dyssynchrony by non-invasive imaging before CRT as a means to predict favorable outcomes, and recent studies have shown an additive prognostic value to QRS width or morphology alone [8-12]. Although echocardiography has been most widely used to measure dyssynchrony, reports of cardiovascular magnetic resonance (CMR) imaging with the use of myocardial tagging have been promising to quantify dyssynchrony by myocardial strain [13-15]. Myocardial tagging has quantitative value, but has not yet gained widespread clinical use, in part because of expertise of specific tagging sequences needed, additional scanning time and the potential for complex post-processing analysis. A more recent semi-automated method called feature tracking (FT-CMR), also using standard clinical steady state free precession (SSFP) CMR images, quantified myocardial strain with high correlation to the labor-intensive myocardial tagging imaging, in a large population with a wide range of cardiac dysfunction [16]. Accordingly, the objective of the present study was to assess the feasibility of utilizing a semi-automated feature tracking CMR software approach applied to routine clinical SSFP imaging to quantify LV radial dyssynchrony in comparison to speckle tracking echocardiography in the same patients.

## Methods

### Patients

We studied 72 consecutive patients who underwent both CMR and echocardiography for the evaluation of LV function, typically on the same day or within a week. All patients were in sinus rhythm. The protocol was approved by the Institutional Review Board for Biomedical Research and patients gave informed consent consistent with this protocol.

### CMR acquisition

CMR was performed with a 1.5 Tesla Magnetom Espree (Siemens Medical Solutions, Erlangen, Germany) with a 32-channel phased array cardiovascular coil. SSFP imaging was acquired during 5 to 10-second breath holds using parallel acquisition acceleration factors (GRAPPA) of 3 (approximately 2 slices per breath hold) and stored digitally for offline analysis. An entire stack of short-axis cine loops was acquired using SSFP imaging with the following typical parameters: echo time 1.22 ms; flip angle, 60°; slice thickness, 6 mm (4 mm gap in short-axis stack); spatial resolution, 1.8 × 1.5 mm; and temporal resolution, 30 frames per RR-interval. Assessment of LV volumes and EF was performed by manual tracing of the endocardial borders at end-diastole and end-systole in each of the short-axis slices using conventional CMR software (Argus, Siemens, Germany). The DICOM formatted files containing LV short-axis images at the mid-ventricular level, using the papillary muscles as an internal anatomic landmark were then exported for subsequent offline post processing analysis of radial dyssynchrony using FT-CMR.

### CMR feature tracking strain analysis

**Figure 1:**
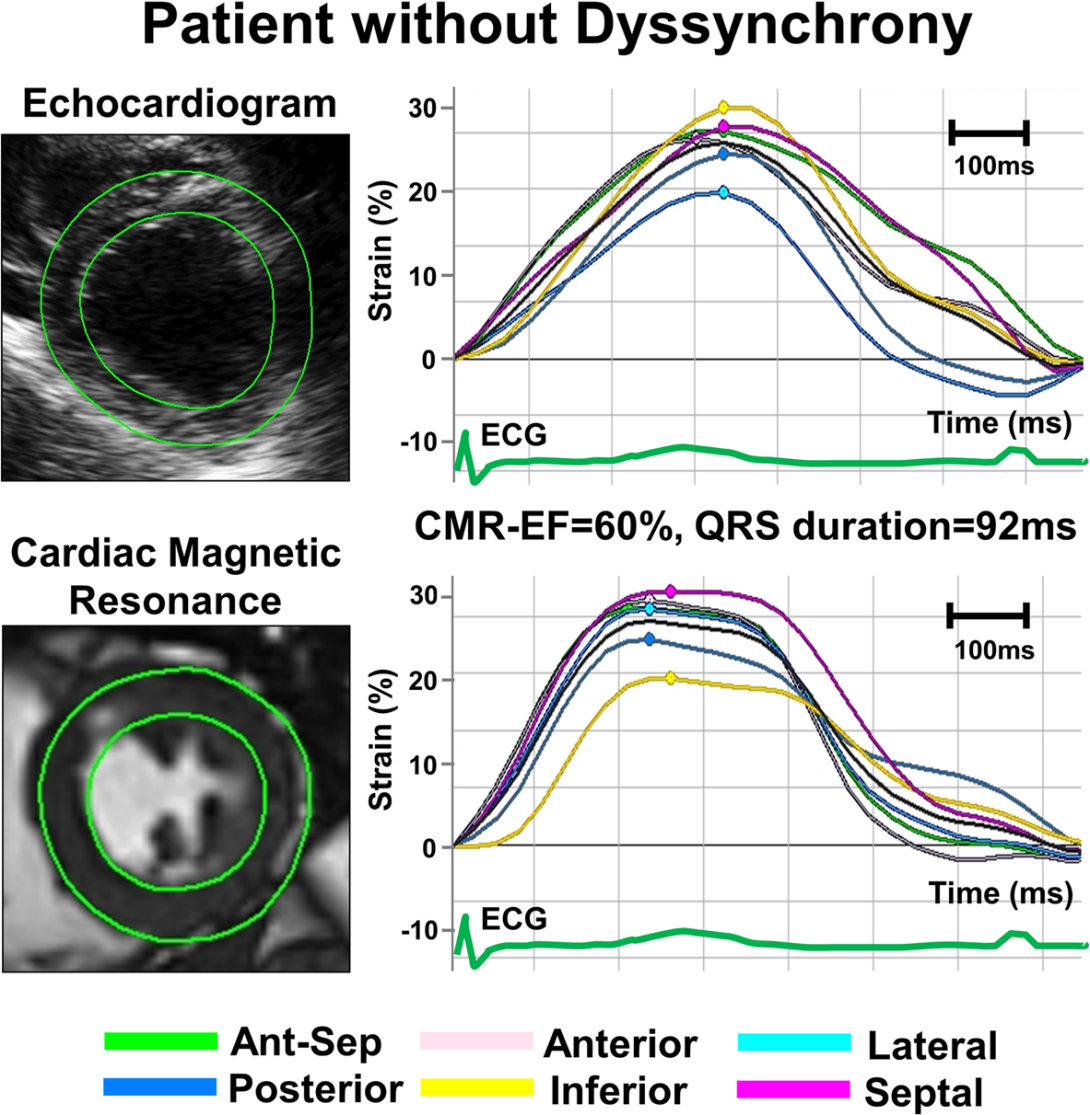
Example of radial time-strain curves by speckle tracking echocardiography (top panel) and feature tracking CMR (bottom panel) in a patient with normal left ventricular (LV) function and without dyssynchrony, demonstrating synchronous time-to-peak-strain curves.

**Figure 2:**
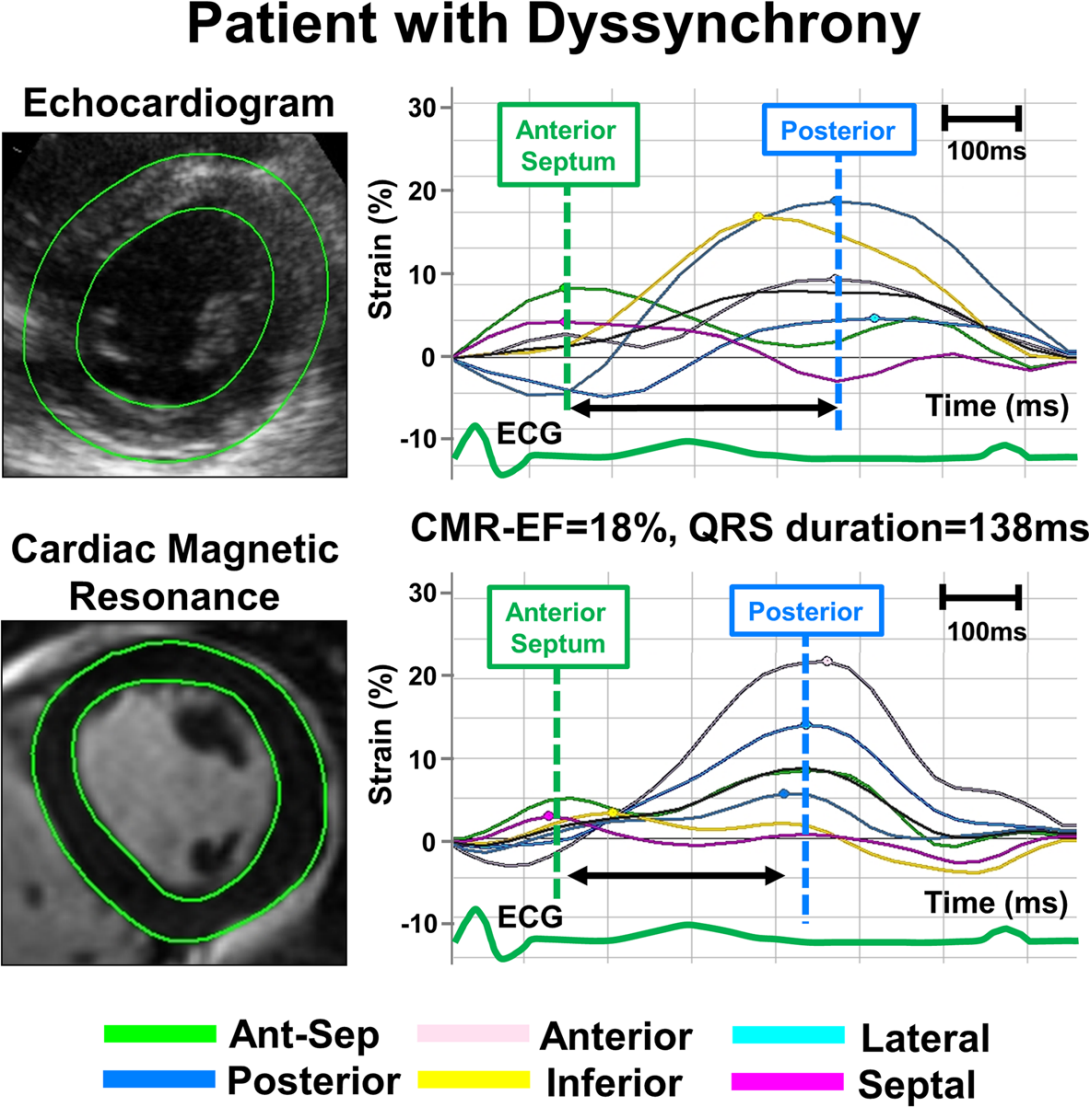
Example of radial time-strain curves by speckle tracking echocardiography (top panel) and feature tracking CMR (bottom panel) in a patient with left ventricular (LV) dyssynchrony, demonstrating dyssynchronous time-to-peak-strain curves with early peak strain in anterior septum (Ant-Sep) segment (green curve) and delayed peak strain in posterior lateral segments (deep blue curve).

A semi-automated feature tracking CMR software (FT-CMR) (2D Cardiac Performance Analysis MR^©^ Version 3.0.0.105, TomTec, Germany) was used as a vector-based analysis tool that based on a hierarchical algorithm that operates at multiple levels using two-dimensional (2-D) feature tracking techniques [16-18]. LV strain was analyzed from routine DICOM data sets by investigators blinded to the clinical, echocardiographic and all other CMR data. A region of interest was traced on the endo- and epi-cardium from a short axis view at the mid-papillary level similar to echocardiographic imaging planes. Adjustment of the region of interest was done after visual assessment during cine loop playback to ensure that the LV segments were tracked appropriately. The color-coded strain curves were extracted from the gray-scale images and were displayed. The entire FT-CMR analytic process required approximately 3 minutes. Radial dyssynchrony was defined as a time difference between the anteroseptal and posterior wall segmental peak strain, and standard deviation (SD) of time to peak strain (Figures 1 and 2).

### Echocardiography acquisition

All echocardiographic studies were performed on commercially available echocardiography systems: Vivid 7 (GE-Vingmed, Horten, Norway) or iE33 (Philips Medical Systems, Andover, Massachusetts). Digital routine grayscale 2-D cine loops from 3 consecutive beats were obtained at end-expiratory apnea from the standard apical 4 and 2-chamber, long axis and mid-LV short-axis views at frame rates of 30 to 100 Hz (mean, 65 ± 15 Hz) as previously described [19]. Gain settings were adjusted for routine clinical grayscale 2-D imaging to optimize endo- and epi-cardial definition. The DICOM formatted files containing LV images were then exported for subsequent offline post processing analysis.

### Echocardiographic speckle tracking strain analysis

Routine B-mode grayscale LV images were analyzed to quantify myocardial strain and volumes (2D Cardiac Performance Analysis^©^ Version 4.3.2.5, TomTec, Munich, Germany) based on stable patterns of natural acoustic markers or speckles within the myocardium, as described previously [20,21]. The analytic process took approximately 3 minutes, similar to FT-CMR analysis. Radial dyssynchrony was defined as the time difference between the anteroseptal and posterior wall segmental peak strain [19] (Figures 1 and 2).

### Statistical analysis

Data were presented as mean ± SD unless otherwise stated. The means were compared with the two-tailed Student’s t-test for paired data. Proportional differences were evaluated with Fisher’s exact test or the Chi-square test. Correlation analysis was performed using linear regression by Pearson’s correlation coefficient while agreement between methods were assessed using Bland-Altman plots [22]. Inter- and intra-observer variability analysis for all feature tracking CMR measurements was performed in 10 randomly selected patients using the identical cine-loop for each view. Inter- and intra-observer variabilities were expressed as the absolute differences divided by the mean value of the measurements. Microsoft Excel (Microsoft Corporation, Redmond, WA, USA) and MedCalc software version 12.0.4.0 (MedCalc Software, Inc., Mariakerke, Belgium) were used. Statistical significance was set at p < 0.05.

## Results

### Patient characteristics

Although FT-CMR analysis was possible in all 72 patients, the study group consisted of 67 patients after 5 patients (7%) were eliminated because it was technically not possible to perform speckle tracking analysis on their suboptimal echocardiography images. Their mean age was 55 ± 15 years, of which 25 were females (35%). QRS width ranged from 68 ms to 202 ms and ejection fraction (EF) ranged from 8% to 78%. the clinical diagnosis in these patients was ischemic heart disease in 20 (28%) including 12 (17%) ischemic cardiomyopathy, non-ischemic disease in 33 (46%) including 17 (24%) idiopathic dilated cardiomyopathy, 4 (6%) hypertrophic cardiomyopathy, 3 (4%) hypertensive heart disease, and 9 (13%) with paroxysmal atrial fibrillation. There were 19 patients (26%) with potential cardiac symptoms, but found to have no evidence of structural heart disease by CMR. According to the QRS duration, 55 patients had narrow QRS (≤ 120 ms) and 17 had wide QRS (> 120 ms). No significant differences were found between patients with narrow and wide QRS duration regarding age, gender and the prevalence of ischemic heart disease. QRS duration, LV end-diastolic volume, LV end-systolic volume, and EF were significantly different between the groups (Table 1).

**Table 1:** Characteristics of patients with wide and narrow QRS

**Variable**	**Patients with wide QRS width** **(≥ 120 ms)**	**Patients with narrow QRS width** **(< 120 ms)**	***P***
Number of patients	55	17	−
Age, yrs	54 ± 15	62 ± 14	0.06
Gender, Male (%)	37 (67%)	10 (59%)	0.07
Ischemic Heart Disease (%)	12 (23%)	8 (47%)	0.09
QRS width, ms	148 ± 24	95 ± 15	< .001
Cardiac magnetic resonance			
LV end-diastolic volume, cm^3^	188 ± 73	259 ± 110	0.003
LV end-systolic volume, cm^3^	103 ± 71	187 ± 98	< 0.001
Ejection fraction,%	49 ± 16	30 ± 17	< 0.001
Feature Tracking CMR Anteroseptal to posterior delay	230 ± 94	77 ± 92	< 0.001
Speckle tracking Echocardiography Anteroseptal to posterior delay	242 ± 101	75 ± 88	< 0.001

*CMR* cardiovascular magnetic resonance, *LV* left ventricular.

### Radial dyssynchrony analysis

**Figure 3:**
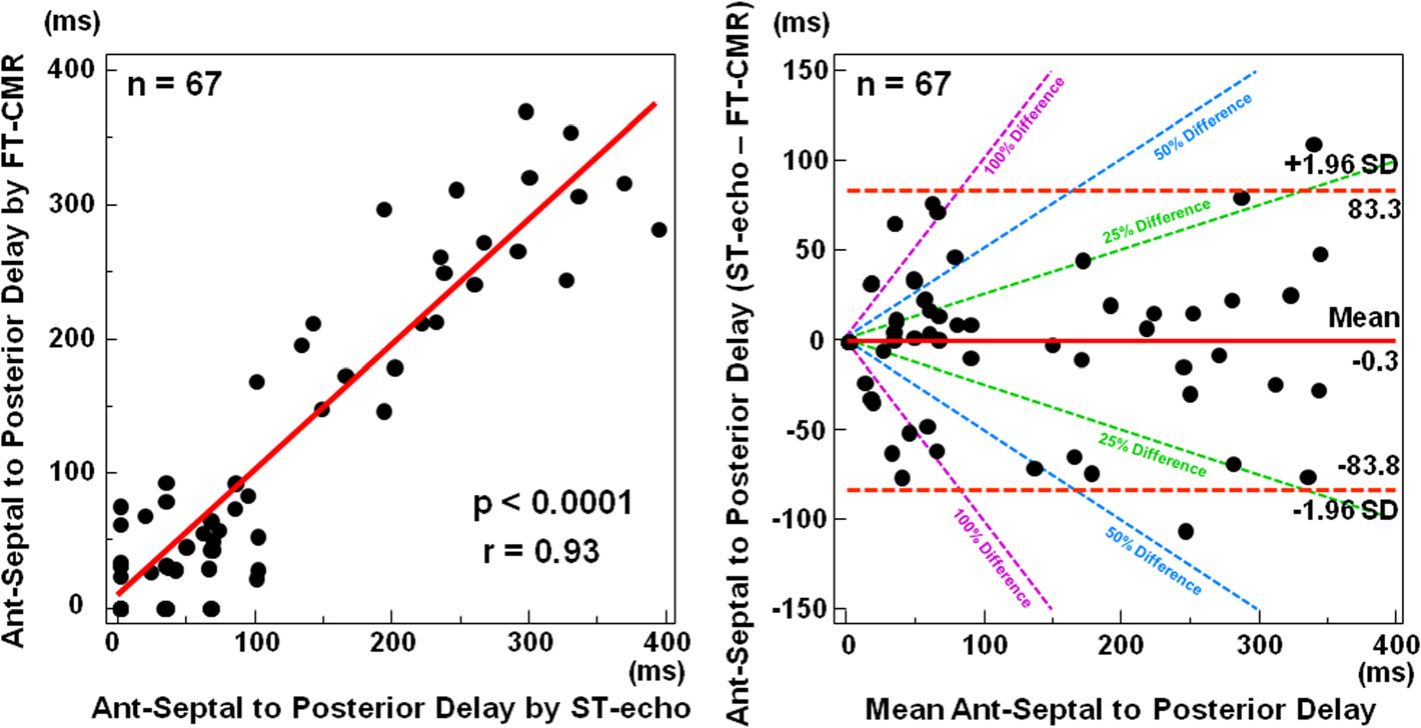
**Regression lines and Bland Altman plots.** Radial dyssynchrony by feature tracking CMR was significantly correlated with that by speckle tracking echocardiography indexes: anteroseptal (ant-sep) to posterior delay by feature tracking CMR versus speckle tracking echocardiography. Bland Altman plot showed limits of agreement between different software for ant-sep to posterior wall delay as well. ST echo = speckle tracking echocardiography, FT CMR = feature tracking CMR.

Imaging data were suitable in 100% (72/72) and 93% (67/72) for quantitative feature tracking CMR and speckle tracking echocardiography radial dyssynchrony analysis, respectively (p = 0.05). Over the relatively wide range of strain delays studied, radial dyssynchrony by FT-CMR correlated with that by speckle tracking echocardiography as follows: anteroseptal to posterior wall delay (r = 0.93, p < 0.0001; Figure 3). The Bland Altman plot showed moderate agreement of feature tracking CMR with speckle tracking echocardiography for anteroseptal to posterior wall delay, but lack of consistent agreement for values less than 100 ms and variability above 200 ms mainly within 25% difference levels (Figure 3). However, anteroseptal to posterior wall delays < 100 ms are seen in normal controls and not of clinical significance with a delay > 130 ms considered as dyssynchronous [9,19]. As expected, radial dyssynchrony was significantly greater in patients with wide QRS than in patients with narrow QRS duration both by feature tracking CMR (anteroseptal to posterior wall delay 230 ± 94 vs 77 ± 92* ms) and by speckle tracking echocardiography (anteroseptal to posterior wall delay 242 ± 101 vs 75 ± 88* ms, all *p < 0.001). Intra- / inter-observer variabilities of feature tracking CMR radial dyssynchrony were respectively 4 ± 4% / 5 ± 6% for anteroseptal to posterior wall delay. Intra- / inter-observer variabilities of speckle tracking echocardiography radial dyssynchrony were respectively 8 ± 9% / 9 ± 10% for anteroseptal to posterior wall delay.

## Discussion

This study introduced the feasibility of more recent FT-CMR software to quantify radial dyssynchrony or routine clinical CMR SSFP cine images. Measures of LV radial dyssynchrony shown to be associated with outcomes after CRT by speckle tracking echocardiography were closely associated with radial dyssynchrony by FT-CMR, at least for the patients with more marked dyssynchrony. In addition, FT-CMR produced similar measures of radial dyssynchrony in patients with narrow and wide QRS duration with at least similar and possibly greater precision as speckle-tracking echocardiography. Although its clinical utility has neither been confirmed nor refuted by the study, FT-CMR might represent an advantageous alternative to the conventional myocardial tagging for the assessment of myocardial strain and dyssynchrony since it does not require availability and expertise of specific tagging sequences, additional scanning time and complex post-processing analysis. In addition, FT-CMR may be applied to patients who have echocardiography images that are unsuitable for speckle tracking analysis.

### Quantification of LV dyssynchrony by echocardiography

Quantification of LV dyssynchrony by non-invasive imaging is of great clinical interest in identifying heart failure patients that could benefit from CRT. A large number of clinical reports using speckle tracking echocardiography have suggested that dyssynchrony analysis was predictive of improved EF response, end-systolic volume reduction and favorable clinical outcome for CRT [8-12,19,20,23-25]. The more recent approach is speckle tracking echocardiography that may be applied to routine gray-scale images and is not limited by Doppler angle of incidence [19,23,25]. Therefore, speckle tracking echocardiography radial strain analysis may permit an accurate quantification of regional wall thickening. According to our previously published prospective and multicenter clinical study known as STAR (speckle tracking and resynchronization), radial dyssynchrony, defined as a time difference in peak septal to posterior wall strain ≥ 130 ms, was associated with probability of unfavorable events after CRT over 3.5 years (p = 0.019) [8]. Two large observational studies of clinical outcomes after CRT have demonstrated that baseline radial dyssynchrony was associated with more favorable long term survival [9,10]. In particular, radial strain dyssynchrony was most predictive of outcomes after CRT in patients whose QRS duration was between 120-150 ms, where clinical response after CRT is less certain by QRS width alone [26]. In addition, baseline radial strain dyssynchrony was associated with clinical outcomes after CRT in patients with non-LBBB QRS morphologies, known to have a more variable response [11]. However, as with any echocardiographic technique, image quality is highly patient and operator dependent. Also, there are limitations to 2D speckle tracking, including need of adequate image quality, out-of-plane speckle motion, software algorithm issues (cross-platform values are not interchangeable), and region-of-interest tracking.

### Quantification of LV dyssynchrony by CMR

CMR may provide an emerging alternative method of quantified dyssynchrony because it is considered to be the gold standard imaging modality for assessment of myocardial strain [13,14,27-29]. CMR allows for high spatial resolution with excellent signal-to-noise ratio and highly reproducible wall motion tracking. Assessment of global and regional myocardial deformation has been feasible with the use of myocardial tagging. In short, it consists of tagging the myocardium with temporary physical markers that can be tracked through the cardiac cycle. This can be done in the image domain, such as saturation, spatial modulation of magnetization SPAMM and complementary SPAMM (CSPAMM) or in the K-space/spatial-frequency domain, such as harmonic phase (HARP), displacement encoding with stimulated echoes (DENSE) [15]. The specific technical details of these sequences have been described elsewhere [15]. Previous investigators have use boundary tracking CMR approaches to assess cardiac dyssynchrony [30,31]. However, CMR measurements of myocardial strain and dyssynchrony has been mostly seen as an important research tool, and have not gained widespread clinical use because they are considered by some to be a cumbersome and complicated image acquisition and post processing analysis. Technological advances in parallel processing, stronger fields gradients and better receiver coil units have allowed improvements in both the spatial and temporal resolution have allowed for the quantification of dyssynchrony without the need of tagged images, using conventional CMR SSFP images [32-34]. Chalil et al, have recently shown that tissue-synchronization imaging derived from SSFP cine images, allowed mechanical dyssynchrony assessment identifying patients with increased risk for major cardiovascular event [32,33]. In this study, we assessed the feasibility of a novel semi-automated feature tracking CMR software (FT-CMR) approach using SSFP imaging. FT-CMR allowed easy quantification of radial dyssynchrony which was highly correlated with dyssynchrony measures by speckle tracking echocardiography (r = 0.93, p < 0.0001). In addition, FT-CMR was able to identify patients with narrow and wide QRS with same precision as conventional speckle-tracking echocardiography, albeit with lower variability of only 4 ± 4% for intra-observer and 5 ± 6% for inter-observer when comparing speckle tracking echocardiography, which had an intra- and inter-observer variability averaging from 8 to 9% and as described previously [19,21]. Lastly, attesting to the superior image quality of CMR, radial dyssynchrony analysis was suitable in all the 72 patients (100%) using quantitative FT-CMR vs. 67 patients (93%) using speckle tracking echocardiography analysis (p = 0.05).

### Clinical implications

The present study demonstrates the feasibility of FT-CMR for identification and quantification of LV dyssynchrony in patients with a wide range of QRS width in a manner similar to speckle tracking echocardiography, at least in patients with greater degrees of dyssynchrony. Given the current controversy of echocardiography diagnosis in LV dyssynchrony, the CMR aided quantification appears to be an alternative approach because of the generally excellent image quality. However, there is little direct comparison to support the conclusion that FT-CMR can be used instead of speckle tracking for patients with dyssynchrony. Since strain analysis using CMR tagging is cumbersome, it may be suggested that a combination of CMR-aided scar quantification plus feature tracking CMR based radial dyssynchrony may provide an invaluable tool as a supplement to speckle tracking echocardiography, or as an alternative in patients being evaluated for CRT with suboptimal image quality.

### Study limitations

One limitation of the present study is that there is no validation with CMR tagging. Previous studies have found that, although the average strain values agree well with CMR tagging, regional FT-CMR may be highly variable [35]. Another limitation is that there are no long term follow-up data on EF and/or clinical response was included in this study. Furthermore myocardial scar burden was not assessed in this study and we cannot infer the relationship and interplay of scar with radial dyssynchrony. Other technical limitation was a relatively slow frame rate of 30 frames per RR-interval (approximately 30–50 frames/sec) for the CMR image acquisition. However, these frame rates were adequate to produce high-quality data that was comparable to speckle-tracking derived radial dyssynchrony. In addition, other protocols allow for high frame rate acquisition. Furthermore, FT-CMR algorithm relies on the combination of speckle-tracking, endocardial tissue-blood border (edge detection) and the periodicity of the cardiac cycle using R-R intervals [36,37]. Phantom analysis has shown that one needs a minimum of 25 frames/sec. Increasing frame rate beyond that will not result in improvement of strain analysis, as the pixel size will be bigger than the frame by frame differences. Therefore, an increase in frame rate will not result in better strain analysis unless is coupled with improvement in the spatial resolution [37]. It is a limitation that no patients with atrial fibrillation were included in this study. Another limitation was that no inter-study reproducibility of the method of dyssynchrony assessment has been attempted, nor has been reported previously, so this remains unknown.

## Conclusions

The semi-automated feature tracking CMR post processing software readily delivers measurements of radial dyssynchrony from routine SSFP imaging. For patients with more marked dyssynchrony, at least, these showed reasonable agreement with those from speckle tracking echocardiography. The FT-CMR method may represent a useful alternative to the conventional myocardial tagging for the assessment of myocardial strain and dyssynchrony since it does not require availability and expertise of specific tagging sequences, additional scanning time and complex post-processing analysis. In addition, CMR-aided scar quantification plus FT-CMR radial dyssynchrony assessment may provide a tool to supplement to speckle tracking echocardiography, or as an alternative in patients with suboptimal acoustic windows being evaluated for CRT. The clinical usefulness of the method, for example in predicting prognosis in CRT patients, remains to be investigated.

## Competing interests

The authors declare that they have no competing interests.

## Authors’ contributions

TO and SKS designed the study protocol, acquired and analyzed the data and drafted the manuscript. DRL, TO, JM, EBS and DS helped acquire and analyze the data and critically revised the manuscript. JLC assisted in interpretation of data and critically revised the manuscript. JG assisted with study design and interpretation of data, critically revised the manuscript and supervised the entire project. All authors read and approved the final manuscript.
